# Enhanced Recovery after Surgery Protocol Accelerates Recovery of Lumbar Disc Herniation among Elderly Patients Undergoing Discectomy via Promoting Gastrointestinal Function

**DOI:** 10.1155/2021/3573460

**Published:** 2021-11-22

**Authors:** Xiaohai Zuo, Linbang Wang, Longzhu He, Pei Li, Dandan Zhou, Yiping Yang

**Affiliations:** ^1^Department of Orthopaedic Surgery, The People's Hospital of Jiulongpo District, Chongqing 400050, China; ^2^Department of Orthopaedic Surgery, The First Affiliated Hospital of Chongqing Medical University, Chongqing 400016, China; ^3^Department of Oral Medicine, The Hospital of the RYTIME DENTAL, Chongqing 400000, China; ^4^Department of Orthopedics, The Third Affiliated Hospital of Chongqing Medical University, Chongqing 401120, China; ^5^Department of Gastroenterology, The People's Hospital of Jiulongpo District, Chongqing 400050, China; ^6^Department of Pain Treatment, The People's Hospital of Jiulongpo District, Chongqing 400050, China

## Abstract

This study aimed to analyze the effect of the enhanced recovery after surgery (ERAS) protocol on the recovery of gastrointestinal function in patients with lumbar disc herniation after discectomy. A total of 179 patients with lumbar disc herniation were randomly divided into the ERAS and non-ERAS groups. The non-ERAS group received routine nursing, and the ERAS group received ERAS strategy. The two groups were compared for general recovery indicators such as postoperative hemoglobin and prealbumin, satisfaction, and length of hospital stay. Gastrointestinal function was also evaluated, such as postoperative feeding time, intestinal chirping recovery time, intestinal exhaust gas recovery time, and complications such as ileus, nausea, and vomiting. The satisfaction of patients in the ERAS group (86.15 ± 2.43) was significantly higher than that in the non-ERAS group (77.19 ± 3.32), and the difference was statistically significant (*P* < 0.05). The average time of eating in the ERAS group was 2.27 h after surgery. In addition, the amount of eating in the ERAS group was significantly better than that in the non-ERAS group, and the difference was statistically significant. In the ERAS group, intestinal chirping recovery time recovered to normal time, and exhaust recovery time and average defecation time were significantly shorter than those in the non-ERAS group. In the ERAS group, the average amount of hemoglobin and prealbumin decreased 3 days after operation, which was significantly lower than that in the non-ERAS group. To sum up, ERAS has an evident effect on the recovery of gastrointestinal function after discectomy of disc herniation, which can promote the recovery of patients.

## 1. Introduction

Lumbar disc herniation in the elderly population may cause significant neural compression, leading to increased pain and poor quality of life of patients. Therefore, identifying effective interventions that could improve the quality of life of elderly patients with lumbar spinal disorders is important [1]. Discectomy has been recognized as a primary treatment of degenerative lumbar spine disorders; however, the surgical stress response, such as immunosuppression, increased catabolism, hypercoagulable states, and free radical production, is associated with major surgery [2]. These physiologic alterations are associated with organ function, which may result in undesirable postoperative complications, pain, and extended convalescence [3].

Postoperative paralytic ileus is a frequent complication after lumbar spinal surgery, with an incidence ranging between 2.6% and 12%, depending on the invasiveness of the complication and approach of the surgery [4]. It leads to increased postoperative morbidity, longer hospital stays, and increased medical costs. Several mechanisms are thought to play a role in postoperative ileus, including sympathetic reflexes, effects of local and systemic inflammatory mediators, and changes of hormone transmitters. Numerous potential treatment options for postoperative ileus have been reported; however, their efficacy is usually limited [5]. In previous reports, lumbar spinal surgery in the aging population has increased [6]. Elderly patients are often complicated with chronic constipation [7]; thus, they may suffer from a higher risk of postoperative ileus after orthopedic surgery than younger patients [8, 9]. Therefore, finding effective measures to prevent postoperative paralytic ileus in the elderly after lumbar spinal surgery is of great significance. Many therapies, including early enteral nutrition, early removal of the nasogastric tube, gastrointestinal motility drugs, and physical therapy, have been suggested and applied in clinical work to prevent postoperative paralytic ileus [10, 11]. However, these therapies cannot be routinely or widely used because of either low compliance or limited clinical efficacy [12].

Enhanced recovery after surgery (ERAS) reduces the surgical stress response, minimizes postoperative complications, and increases readmission rates [2], which are important for vulnerable patients, who often suffer from comorbidities, and the elderly [13]. ERAS can also improve the gastrointestinal function of postoperative orthopedic patients, such as decreasing postoperative ileus, nausea, and vomiting, among which postoperative ileus is a common complication of discectomy and is estimated to occur in a considerable proportion of patients undergoing surgery [14]. Livingston and Passaro defined ileus as “the functional inhibition of propulsive bowel activity, irrespective of pathogenetic mechanism.” [15] The pathogenesis of ileus is multifactorial with immobility, opioids, and anesthesia, which affect bowel function [16]. Studies have demonstrated that postoperative ileus can increase the length of hospital stay (LOS) and costs significantly [17]. This study aimed to evaluate the impact of ERAS on gastrointestinal function among elderly patients with spinal disorders undergoing surgery.

## 2. Methods

### 2.1. Inclusion and Exclusion Criteria

This is a retrospective cohort study. The study protocol was approved by the Ethics Committee for Human Subjects of the People's Hospital of Jiulongpo District. Written informed consent was obtained from each patient. Patient data were anonymized in this study. Altogether, 179 patients with lumbar disk herniation over the age of 65 who underwent posterior lumbar discectomy at two or lower levels from January 2019 to December 2020 were assigned to the non-ERAS group (*n* = 95) and the ERAS group (*n* = 84). Details of the enrolled patients could be found in Supplementary table. All the treatments were conducted by the same surgical team. Patients in the non-ERAS group were treated under traditional perioperative protocols. Diagnosis of lumbar disk herniation was conducted by at least two spinal orthopedic specialists based on MRI images of the lumbar spine and clinical symptoms, and the responsibility segments were identified. Patients who had typical spinal stenosis symptoms and did not respond to conservative treatments were indicated for surgery. Individuals who had neoplasm, cauda equina injury, trauma, and infectious disease were excluded from this study. All data were collected from the electronic medical record. Demographic data included gender, age, and body mass index (BMI). Comorbidities included hypertension, heart disease, diabetes, osteoporosis, stomach problem, bowel or intestinal problem, and psychological symptoms. Other indices included the American Society of Anesthesiologists (ASA) physical status score, preoperative Japanese Orthopaedic Association (JOA) Score, Oswestry Disability Index (ODI), and visual analogue scale (VAS) for the back and leg. Operative records used for analysis included the number of fusion levels, operative time, and intraoperative blood loss. The primary outcome data included complications, postoperative pain scores, LOS, and 30-day readmission rates.

### 2.2. ERAS Interventions

**Figure 1:**
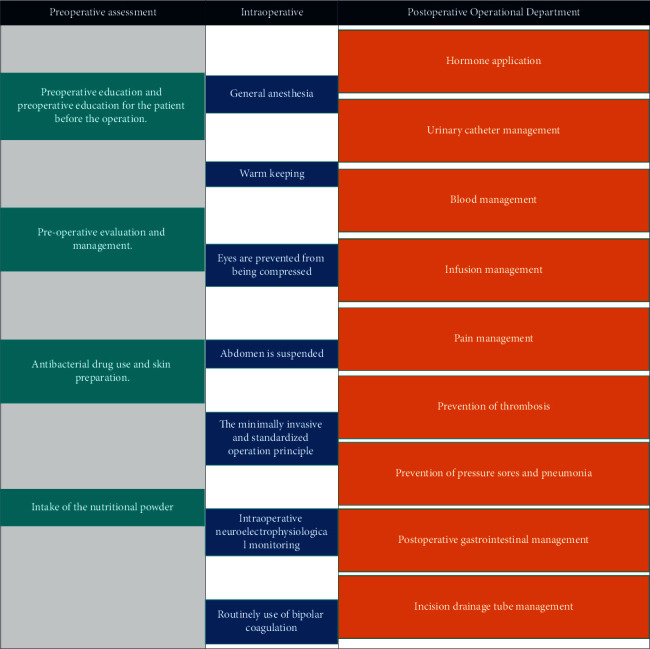
Summary of conducted perioperative topics for ERAS with discectomy.

In this study, we followed the methods of Wang et al. [18]. The ERAS program was proposed and planned by a core group of anesthesiologists, nutritionists, spine surgeons, physicians, physical therapists, nurses, and geriatricians after literature review and experience exchange [19–21]. With the approval of the Ethical Committee for Human Subjects of the People's Hospital of the Jiulongpo District, the implementation of the ERAS program began in June 2019. ERAS interventions were divided into preoperative, intraoperative, and postoperative, including the following administration: (1) patient education and counseling, (2) antibiosis before surgery, (3) preoperative fasting (without drinks 2 h and food 4 h before surgery), (4) multimodal analgesia, (5) standard anesthetic protocol, (6) gastrointestinal management, (7) early feeding after surgery, (8) early mobilization medical, (9) early removal of the bladder catheter, and (10) antithrombotic prophylaxis. Details of ERAS are displayed in Figure 1.

### 2.3. Statistical Analysis

Statistical analyses were performed by GraphPad software (version 8.0). Student's *t*-test and *χ*^2^ test were used to compare comorbidity data, patient demographics, clinical results, and baseline health indicators among the groups. We also used multivariate linear regression analysis and multivariable logistic regression to assess the association among the risk factors of ERAS elements and ileus rate. Differences were considered significant at a level of *P* value less than 0.05.

## 3. Results

### 3.1. Demographics

**Figure 2:**
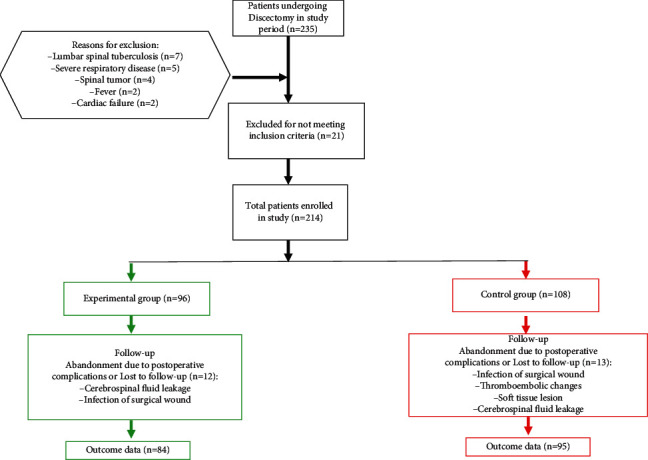
Flow of patients through the study.

**Figure 3:**
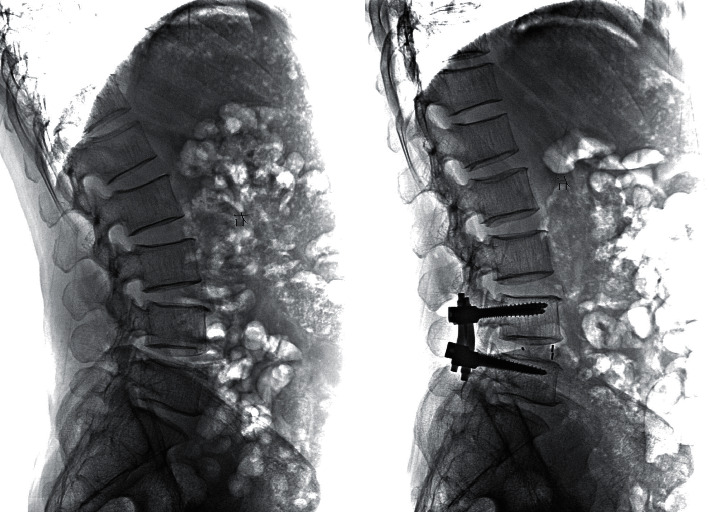
Representative case of a patient with an L4-5 LDH. Preoperative (a) and postoperative (b) radiographs were obtained by ERAS during the operation.

A total of 179 patients (Figure 2) were included, with 84 patients in the ERAS group (46 men and 38 women, mean age: 71.31 ± 9.17 years, mean BMI: 24.17 ± 2.96) and 95 patients in the non-ERAS group (51 men and 44 women, mean age: 71.63 ± 9.01 years, mean BMI: 24.75 ± 3.67). All surgeries were performed by a senior surgeon (Figure 3). Preoperative characteristics were similar between the two groups (Table 1). Demographic data were compared, and no statistically significant differences were observed between the two groups. In addition, no significant differences were noted in comorbidities, ASA grade, or the number of fusion levels between both groups. The mean operative time and intraoperative blood loss in the ERAS and non-ERAS groups showed no significant difference. Moreover, the mean preoperative JOA, VAS for the back and legs, and ODI score showed no significant difference (Table 1).

### 3.2. Compliance with the ERAS Protocol

Our ERAS protocol included 14 pathways, and the overall pathway compliance was 96.4% (Table 2). Patient education and counseling, no prolonged fasting, antimicrobial prophylaxis, and all intraoperative ERAS interventions were performed in all patients of the ERAS group. The pathway with the lowest compliance was early oral feeding (Table 2).

### 3.3. Outcomes

The main clinical outcomes are shown in Table 3. After the implementation of ERAS, no significant difference in 30-day readmission and mortality was found between the ERAS group and the non-ERAS group. Furthermore, the mean postoperative VAS for the back and legs showed no significant difference at 30-day follow-up as complete data were available for 83% of patients at this early time point. However, we observed a statistically significant decrease in LOS in the ERAS group (11.27 ± 4.07 days in the ERAS group versus 14.60 ± 2.13 days in the non-ERAS group, *P* < 0.05). The patient satisfaction rate of the ERAS group was 92.00%, and the difference was statistically significant (*P* < 0.05). In the ERAS group, the average time of eating was 2.27 h after surgery, and patients consumed much more food than those of the non-ERAS group. Moreover, the time for bowel sounds to return to normal (3–5 times/min) was 5.63 h; the recovery time of exhaust gas was 8.14 ± 6.52 h, and the average time of defecation was 1.02 days, which were both significantly less than those of the non-ERAS group. The rate of nausea, vomiting, and flatulence complications in the ERAS group was 2.39%, which was less than that of the non-ERAS group, but the difference was not significant. The average amount of hemoglobin in the ERAS group 3 days after operation was 8.14 g, and the average decreased amount of prealbumin was 5.28 g, with statistical significance (*P* < 0.05).

Multivariable linear regression showed that comorbidities (*P* = 0.021), dose of sufentanil (*P*=0.042), operative time (*P*=0.041), and implementation of the ERAS program (*P*=0.036) were significantly correlated with postoperative ileus. On the contrary, age, gender, BMI, smoking history, ASA ≥3, fusion number, blood loss, preoperative VAS for the back, and preoperative VAS for the leg were not related to postoperative ileus. Multivariable logistic regression showed that no characteristics were associated with postoperative ileus (Table 4).

## 4. Discussion

Disk herniation and the loss of disk height are largely associated with aging, which places extra loads on adjacent segments and facet joints, leading to low back pain (LBP). LBP and sciatica can significantly impair patients' psychosocial function, leading to depressive symptoms and sleep disorders. Furthermore, LBP and sciatica are correlated with coronary heart disease in elderly people [22]. However, comorbidities and poor physical function can cause high rates of perioperative complications, such as inpatient morbidity, during lumbar spinal surgery in elderly patients [23, 24]. It is reported that thoracic epidural anesthetics can reduce the duration of postoperative ileus by blocking the nerve reflex of the spinal cord and reducing the use of postoperative anesthesia in patients. Nonsteroidal anti-inflammatory drugs can also accelerate the recovery of intestinal function by inhibiting intestinal inflammation and reducing the use of opioids. Thus, a multimodal treatment approach that combines multiple therapies may be a logical approach [9]. As proposed by Henrik Kehle, a Danish surgeon, ERAS is a multidisciplinary and multiprofessional approach for postoperative patients to obtain a relatively rapid recovery [25]. To date, the basic principles of ERAS have been adopted by surgical specialties in multiple fields [26, 27]. This protocol has been shown to be beneficial particularly for elderly people who have comorbidities or a higher risk of surgical complications. The ERAS protocol is specifically designed for patients in adapting to surgical stresses such as immobility, dehydration, and inflammation by all-encompassing approaches, which focuses on various aspects of perioperative care, including changes in mobilization, fasting, early postoperative oral intake, goal-directed fluids, and multimodal analgesia [28, 29]. In our study, the ileus rate in the cohort of patients in the ERAS group was significantly decreased. In addition, the patients in the ERAS group had a shorter hospital stay and decreased readmission rate.

Shortening the time of fasting and feeding is an important preoperative aspect in our ERAS program [30]. Traditional preoperative fasting time lasting for at least 8 h and oral feeding on postoperative day 1 may cause metabolic stress and insulin resistance caused by inflammatory cytokine release and lipid product accumulation in skeletal muscles and then increase the rate of postoperative complications [31–33]. Therefore, shortening the time of preoperative fasting and postoperative eating can decrease insulin resistance and improve patient comfort [34]. However, research concerning the shortening of postoperative eating time and preoperative fasting time among elderly patients with lumbar surgery is lacking, although studies have indicated that this approach is effective and safe [35]. Our studies have illustrated that oral carbohydrate drink 1.5 h before anesthesia induction and early feeding 5 h after surgery are safe and are not associated with the increasing risk of complications in elderly patients.

At present, the treatment for postoperative ileus is primarily divided into four parts: perioperative prevention, traditional treatment, drug intervention, and surgical treatment [36]. Traditional treatments, including nasogastric decompression, electrolyte replacement, and early bed movement, have poor patient compliance and efficacy [37]. Pharmacological interventions are commonly applied for the prevention of ileus after abdominal surgery, such as motility agents and antiemetics, *μ*-receptor antagonists, and neostigmine; however, efficacy of these interventions is also unsatisfactory [38, 39]. Surgical treatment is only suitable for severe complications caused by intestinal obstruction, such as ischemia or bowel perforation. Therefore, postoperative prevention is crucial in the management of postoperative ileus.

ERAS protocol decreases postoperative ileus rate through multiple mechanisms. Preoperatively, patients are allowed to drink clear fluids prior to surgery up to 2 h in this protocol, which prevents prior-surgery dehydration and allows the intake of preoperative carbohydrate. As reported by Varadhan and Lobo, fluid overload is related to increased bowel edema rates, which leads to ileus [31]. However, maintaining adequate tissue perfusion and intravascular volume is necessary [40]. Thus, fluid administration protocol ERAS aims to maintain intravascular volume and mitigate risks. In our cohort, a significant decrease of intraoperative intravenous fluid (IVF) administration was found in ERAS patients compared with controls. Moreover, the standard hourly volume of IVFs in ERAS patients was decreased drastically. Intraoperatively, we have discovered that the use of sufentanil is associated with the increasing rates of ileus [35]. Sufentanil is known for its inhibitory effects on peristalsis of the gastrointestinal smooth muscle and intestinal motility in rats. In addition, narcotics could activate *μ*-opioid receptors and cause gut motility inhibition, leading to increased ileus rates. Thus, decreasing the use of narcotics plays a vital role in reducing ileus rates. As shown in considerable research, chewing gum is an efficient way to reduce postoperative ileus in the postoperative stage [32]. In our study, the patients in the ERAS group were allowed to chew gums after surgery, which is considered a crucial factor for the significantly decreased rate of ileus in ERAS patients. In the ERAS regimen, chewing gum is a form of sham feeding that can stimulate human intestinal motility [41]. Several possible physiological mechanisms are identified: first, chewing gum stimulates the oropharyngeal chemical mechanoreceptors, activates the cephalovagal pathway, and increases the secretion of gastrointestinal hormones such as motilin, gastric acid, gastrin, and pepsinogen, thus promoting gastrointestinal motility [42, 43]. Second, mastication can stimulate the vagus pathway and increase the release of acetylcholine transmitters, which then bind to nicotine receptors of inflammatory cells, thereby reducing the release of proinflammatory factors and promoting the recovery of gastrointestinal motility [44].

Our results suggest that the ERAS regimen promotes recovery of intestinal function after lumbar surgery in elderly patients, with a significantly accelerated time of first flatus and first defecation. Compared with abdominal surgery, patients in both groups showed significantly better bowel movement. These findings can be explained as follows: first, the operative time of lumbar fusion is relatively short (less than 3 h). Second, the intestinal tract is almost uninterfered during posterior lumbar surgery.

## 5. Conclusions

This study shows the potential application of a practical ERAS protocol in elderly patients after discectomy, which has been proven to decrease LOS and postoperative ileus rate in elderly patients. Further studies with modified approaches are required to improve adherence to the outcomes.

## Figures and Tables

**Table 1:** Patient demographics.

Patient demographics	ERAS	Non-ERAS	*P*
Sample size	84	95	
Age (years)	71.31 ± 9.17	71.63 ± 9.01	0.50
Male/female	46/38	51/44	1
Body mass index	24.17 ± 2.96	24.75 ± 3.67	0.86
Smoker	6	7	1
Comorbidities			
** **Hypertension	53	49	0.13
** **Heart disease	17	15	0.56
** **Chronic lung disease	1	2	1
** **Diabetes	16	13	0.42
** **Osteoporosis	11	9	0.48
** **Gastrointestinal	6	7	1
** **Psychological symptoms	1	0	0.47
Preoperative JOA	7.30 ± 3.07	6.99 ± 2.97	0.49
Preoperative ODI, %	60.88 ± 8.31	61.63 ± 9.27	0.57
Preoperative VAS (back)	6.48 ± 1.21	6.75 ± 1.01	0.11
Preoperative VAS (leg)	6.19 ± 1.76	6.34 ± 1.88	0.58
ASA grade			
** **I	11	11	
** **II	60	60	
** **III	13	13	
** **IV	0	0	
No. of fusion levels			
** **1	62	67	0.74
** **2	22	28	0.74
Operative time (min)	163.88 ± 49.23	168.43 ± 51.62	0.55
Intraoperative blood loss (ml)	283.63 ± 169.64	243.63 ± 188.64	0.14

**Table 2:** ERAS pathway compliance.

Compliance with the ERAS program	
Variable	*n* (%)
Preoperative ERAS items	
** **Patient education and counseling	84 (100)
** **No prolonged fasting	84 (100)
** **Fluid and carbohydrate loading	84 (100)
** **Antithrombotic stockings	84 (100)
** **Antimicrobial prophylaxis	84 (100)
Intraoperative ERAS items	
** **Tranexamic acid	84 (100)
** **Maintenance of normothermia	84 (100)
** **Local infiltration analgesia	84 (100)
** **Fluid balance	84 (100)
Postoperative ERAS items	
** **Early ambulation	77 (91.7)
** **Early removal of the bladder catheter	67 (79.8)
** **Early oral feeding	63 (75)
** **Chewing gum	80 (95.2)
** **Intermittent pneumatic compression	82 (97.6)
** **Overall compliance (rate)	81 (96.4)

**Table 3:** Postoperative outcomes.

Outcome measure	ERAS	Non-ERAS	*P*
LOS^*∗∗∗*^	11.27 ± 4.07	14.60 ± 2.13	0
30-day readmission	1	2	0.47
30-day mortality	0	0	1
Decreased amount of hemoglobin (g/L)^*∗∗∗*^	8.14 ± 2.06	12.37 ± 2.21	0
Decreased amount of prealbumin (g)^*∗∗∗*^	5.28 ± 1.07	8.32 ± 1.40	0
Postoperative time (days)^*∗∗∗*^	6.14 ± 1.24	8.14 ± 2.38	0
Satisfaction^*∗∗∗*^	86.15 ± 2.43	77.19 ± 3.32	0
Preoperative VAS (back)	7.09 ± 0.83	7.04 ± 0.67	0.66
Preoperative VAS (legs)	7.32 ± 0.72	7.44 ± 0.23	0.13
Gastrointestinal indicators			
** **Ileus rate^*∗∗∗*^	5.89	31.89	0
** **Postoperative feeding time (h)^*∗∗∗*^	2.27 ± 1.50	4.14 ± 3.92	0
** **Food intake (h)^*∗∗∗*^	5.58 ± 2.57	3.52 ± 2.43	0
** **Borborygmus recovery time (h)^*∗∗∗*^	5.63 ± 2.54	6.02 ± 3.51	0.04
** **Intestinal exhaust gas recovery time (h)	8.14 ± 6.52	10.21 ± 7.16	0.05
** **Postoperative defecation time (d)^*∗∗∗*^	1.02 ± 1.28	2.31 ± 2.10	0
** **Postoperative nausea and vomiting	2.39	9.53	0.06
General complications			
** **Cerebrovascular accident	0	1	1
** **Surgical site infection	1	3	0.62
** **Spinal fluid leakage	2	3	1
** **Neurological	1	2	1
** **Deep vein thrombosis	0	1	1
** **Cardiac arrest	0	0	1

^∗^ *P* value less than 0.05; ^∗∗^*P* value less than 0.01; ^∗∗∗^*P* value less than 0.001.

**Table 4:** Multivariable analyses for LOS and complications.

Characteristics	Multivariable linear regression for LOS		Multivariable logistic regression for any complications	
	Coefficient (95% CI)	*P* value	OR (95% CI)	*P* value
Age	0.25 (−0.12 to 0.27)	0.35	1.09 (0.87–1.28)	0.49
Female	1.12 (−0.47 to 1.22)	0.10	1.09 (0.93–1.17)	0.24
BMI	−0.023 (−0.33 to 0.11)	0.74	0.94 (0.89–1.02)	0.07
Smoker	0.78 (−0.19 to 1.20)	0.15	2.21 (0.84–3.12)	0.14
Comorbidities	1.24 (0.23 to 1.63)	0.02	1.46 (0.87–2.21)	0.06
Fusion number	2.21 (−1.19 to 2.97)	0.18	1.99 (0.98–2.38)	0.11
Estimated blood loss	1.21 (−1.96 to 3.75)	0.07	1.74 (0.35–2.06)	0.88
Intraoperative fluids	0.78 (0.01 to 1.17)	0.65	2.11 (0.85–2.21)	0.10
Dose of sufentanil^*∗*^	0.98 (0.53 to 1.71)	0.04	1.62 (0.99–1.72)	0.05
Operative time^*∗*^	0.41 (−0.02 to 0.91)	0.04	0.93 (0.87–3.26)	0.13
ERAS^*∗*^	0.94 (0.73 to 1.13)	0.04	1.23 (0.79–1.88)	0.06
Preoperative VAS (back)	0.29 (−0.56 to 0.98)	0.36	0.71 (0.65–1.46)	0.22
Preoperative VAS (leg)	0.75 (−0.60 to 2.11)	0.75	1.22 (0.91–2.13)	0.34
Preoperative ODI (%)	−0.01 (−0.08 to 0.21)	0.38	1.26 (0.64–2.48)	0.31

^∗^ *P* value less than 0.05; ^∗∗^*P* value less than 0.01; ^∗∗∗^*P* value less than 0.001.

## Data Availability

The data used to support the findings of this study are available from the corresponding author upon request.

## Supplementary Materials

Supplementary Materials Supplementary table: the detailed data of the enrolled patients.
