# Supplementary material for: Enhanced Recovery after Surgery Protocol Accelerates Recovery of Lumbar Disc Herniation among Elderly Patients Undergoing Discectomy via Promoting Gastrointestinal Function
Source: Pain Res Manag. 2021 Nov 22;2021:3573460. doi: 10.1155/2021/3573460 (PMC8629654; doi:10.1155/2021/3573460)
Supplement: Supplementary Materials — Supplementary table: the detailed data of the enrolled patients. [file 3573460.f1.pdf]

Number	Age (year)	Sex (1=male, 2=female)	BMI	Smoler	Fusion number	Estimated blood loss	Intraoperative fluids	Dose of sufentanil	Operative time (min)	ERAS (1=yes, 2=no)	Satisfaction score	VASback	VASleg	ODI%	LOS	Hemoglobin decreased amount (g)	Pre-albumin decreased amount (g)	Intestinal exhaust gas recovery time (h)	Postoperative defecation time (d)	Intestinal chirping recovery time (h)	Postoperative feeding time (h)
1	65	1	22.3	1	1	150	1700	30	115	1	98	6	4	62	6	22	24	8.2	1	11.2	2.8
2	67	1	23.2	1	1	200	1100	40	125	1	85	5	5	42	6	22	24	8.4	1	11.2	2.9
3	82	1	27.3	2	1	50	1100	40	110	1	96	2	4	40	6	22	24	8.8	1	11.4	2.9
4	65	1	27.1	1	1	300	2700	40	125	1	88	2	7	68	6	22	25	9	1	11.6	2.9
5	79	1	21.5	1	2	100	1100	40	105	1	67	8	5	81	6	23	25	9	1	12	3.1
6	79	1	21.5	2	2	200	1700	40	105	1	95	5	8	75	6	23	25	10.2	1	12.2	3.2
7	70	1	25.8	1	1	100	1600	40	125	1	88	6	6	68	6	23	25	10.7	1	12.2	3.2
8	73	1	29.6	1	2	200	1100	40	105	1	84	5	5	53	6	23	25	11	1	12.2	3.2
9	69	2	24.5	1	1	100	1700	45	135	1	71	5	9	86	6	24	26	11.2	1	12.3	3.2
10	67	1	25.3	1	1	150	1100	40	165	1	79	8	7	78	6	24	26	12	2	16.3	2.6
11	65	2	22	1	1	50	1200	35	105	1	94	5	5	36	6	25	5	11.2	1	12.3	3.2
12	67	1	23.7	1	2	200	1750	45	194	1	90	5	2	50	6	25	6	12	2	16.4	2.7
13	74	2	23.2	1	1	50	1200	35	120	1	92	4	5	41	6	25	11	11.6	1	11.2	2.8
14	71	1	27.3	1	1	100	1600	40	170	1	77	7	7	40	6	25	12	12.1	2	16.5	2.7
15	69	1	24.8	1	1	50	2200	35	120	1	97	5	6	70	6	27	14.2	11.6	1	11.2	2.9
16	66	1	27	2	1	100	1700	40	165	1	82	4	5	36	6	27	16	12.2	2	16.5	2.8
17	67	2	26.4	1	1	100	1700	45	135	1	81	6	4	40	7	27	17	11.6	1	11.4	2.9
18	66	2	20.5	1	1	50	720	45	135	1	94	5	9	95	7	28	17	12	1	11.6	2.9
19	77	2	22.9	1	1	100	1300	50	165	1	78	4	8	65	6	28	18	12.3	2	16.5	2.8
20	67	2	24.1	1	1	200	2200	45	155	1	67	5	5	35	7	28	19	12	1	12	3.1
21	73	1	23.9	1	1	150	1600	40	125	1	73	6	8	72	7	30	42	12.1	1	12.2	3.2
22	71	2	23.2	1	2	150	2700	50	200	1	92	8	1	70	6	31	42	12.3	2	16.5	2.9
23	71	1	26	1	1	50	1100	40	115	1	84	6	0	34	7	31	43	12.2	1	12.2	3.2
24	68	1	26.7	1	1	50	1600	30	117	1	82	2	4	52	7	31	26	12.3	1	12.2	3.2
25	65	1	20.5	2	1	50	1600	40	170	1	93	6	6	56	6	31	26	12.3	2	16.5	2.9
26	68	2	28.3	1	1	200	2000	45	135	1	97	5	4	36	7	31	26	12.3	1	12.3	3.2
27	65	2	23.6	1	1	100	1600	35	120	1	87	5	8	70	7	34	30	12.3	1	12.3	3.2
28	72	1	27.7	1	2	200	2100	45	195	1	75	4	5	44	6	35	31	12.4	2	16.5	2.9
29	72	1	22.6	1	2	200	2200	40	105	1	80	6	6	59	7	35	31	12.4	1	12.3	3.2
30	67	1	23.2	1	1	100	1600	40	125	1	97	4	2	33	7	35	31	12.5	1	12.4	3.2
31	76	2	28.1	1	1	200	2200	40	125	1	82	3	6	55	7	35	31	12.6	1	12.4	3.2
32	73	1	23.8	1	2	200	2700	45	195	1	93	5	7	56	6	35	32	12.5	2	16.5	3.1
33	76	1	20.7	1	1	100	1700	40	110	1	98	7	8	75	7	36	32	12.6	1	11	2.8
34	73	1	26.7	1	1	100	2300	40	125	1	92	8	4	79	7	36	33	12.6	1	11.2	2.8
35	76	1	28.3	2	2	50	1200	50	195	1	82	7	8	81	6	36	33	12.6	2	16.5	3.2
36	77	1	23.4	2	1	100	1100	40	115	1	88	5	6	50	3	3	34	12.7	1	11.2	2.9
37	74	2	24	1	1	100	1700	35	120	1	96	4	9	91	3	5	34	12.7	1	11.4	2.9
38	66	2	26.7	1	2	300	2600	65	185	1	70	8	6	75	6	6	34	12.6	2	16.5	3.2
39	86	1	26	1	2	200	2200	45	150	1	97	4	6	49	6	7	34	12.6	2	16.5	3.2
40	72	2	22.6	1	2	600	2820	40	163	1	65	5	7	60	3	7	34	12.7	1	11.6	2.9
41	65	1	24	2	1	100	1700	40	123	1	97	7	5	76	3	8	34	12.8	1	12	3.1
42	72	2	21.3	1	2	100	1700	40	105	1	89	2	5	46	3	9	36	13.2	1	12.2	3.2
43	76	2	27.1	1	1	50	1700	35	105	1	69	9	8	83	4	12	37	13.3	1	12.2	3.2
44	74	2	22.9	1	1	100	1700	35	102	1	86	5	4	36	4	14	49	13.3	1	12.2	3.2
45	65	2	24	1	1	100	2200	40	130	1	98	5	9	75	4	15	52	13.5	1	12.3	3.2
46	74	2	25.6	1	2	150	3300	50	205	1	91	6	4	50	6	17	52	12.7	2	16.6	3.2
47	65	1	28.7	1	1	100	1600	40	110	1	65	2	6	62	4	19	53	14.3	1	12.3	3.2
48	68	2	23.4	1	1	200	2200	35	102	1	90	8	8	79	4	19	53	15.2	1	12.3	3.2
49	73	1	24.8	1	1	300	2800	40	115	1	86	6	5	80	4	19	53	15.2	1	12.4	3.2
50	73	1	20.7	1	1	50	1700	30	120	1	85	4	9	88	4	20	53	15.2	1	12.4	3.2
51	73	2	29.3	1	1	100	2600	50	165	1	83	4	5	41	7	20	53	12.7	2	16.6	3.2
52	71	2	23.2	1	1	100	1600	45	134	1	69	5	8	78	4	20	53	15.3	1	12.4	2.6
53	73	2	16.8	1	1	50	1600	35	120	1	79	8	6	76	4	21	54	15.3	1	12.5	2.6
54	66	2	24.8	1	1	50	1700	35	121	1	69	8	1	79	4	21	54	15.4	1	12.5	2.6
55	75	2	23.3	1	1	300	1200	40	125	1	86	4	4	38	4	21	48	15.4	1	12.6	2.6
56	76	2	27.1	1	2	200	1800	50	205	1	92	1	5	40	7	22	49	12.7	2	16.7	3.2
57	67	2	18.9	1	1	50	1200	35	120	1	91	8	8	72	4	22	49	15.4	2	12.6	2.6
58	66	1	23.8	1	1	100	1600	40	110	1	97	6	6	39	4	22	52	15.4	2	13.2	2.6
59	70	2	24.8	1	1	100	1600	35	98	1	94	8	10	98	4	22	43	15.4	2	13.2	2.6
60	79	1	20.7	1	1	100	1700	35	120	1	92	5	6	52	4	23	44	15.4	2	14.4	2.3
61	85	1	27.7	1	2	200	1700	50	196	1	85	9	6	92	7	23	44	12.8	2	16.7	3.2
62	69	1	26	1	1	100	1300	30	118	1	80	6	6	60	4	23	45	15.5	2	14.5	2.3
63	65	1	24.8	1	1	100	1100	30	116	1	96	6	5	55	4	23	47	15.5	2	14.5	3.3
64	65	2	26	1	1	200	2200	45	135	1	75	1	8	76	4	24	48	4.3	2	14.8	3.3
65	72	2	16.8	1	1	100	1100	35	100	1	69	2	2	15	4	24	49	5	2	14.9	3.3
66	68	1	23.9	1	1	100	1200	30	118	1	91	2	5	40	4	25	49	6	2	15	3.3
67	78	2	23.2	1	2	100	2100	40	108	1	97	5	2	43	4	25	52	6.2	2	15	3.4
68	73	2	27.8	1	2	200	2300	40	160	1	69	7	5	60	4	25	52	6.2	2	15.2	3.4
69	81	2	20.5	1	2	100	1200	65	185	1	90	2	8	70	7	25	53	13.2	2	16.7	3.2
70	66	2	27.5	1	1	50	1700	40	105	1	97	2	9	81	5	27	53	6.5	2	15.2	3.4
71	75	1	22.9	1	2	400	2200	40	105	1	69	6	6	76	5	27	53	7.5	2	15.6	4.2
72	72	2	25.1	1	1	150	2200	40	130	1	92	8	5	74	5	27	53	7.9	2	15.6	4.2
73	70	2	22.7	1	1	200	1100	40	125	1	93	0	4	15	5	27	53	8	2	15.6	4.2
74	71	2	26.1	1	1	150	1600	40	127	1	83	5	6	59	5	27	55	8	2	16	4.3
75	76	1	19	1	1	100	1700	40	125	1	95	8	8	78	5	27	55	8	2	16	4.3
76	73	1	18	1	2	100	2200	50	196	1	91	6	4	55	7	28	56	15.4	2	16.8	5
77	73	2	22.2	1	1	200	2200	40	130	1	100	6	9	82	5	28	57	8	2	16.2	4.3
78	67	2	24.2	1	2	700	3000	40	110	1	86	2	8	71	5	28	56	8	2	16.2	4.3
79	76	1	20.5	1	1	200	1700	40	123	1	98	5	7	75	5	28	57	8.2	2	16.2	2.5
80	66	1	30.4	1	2	200	1700	40	105	1	93	3	2	22	5	28	57	8.4	2	16.2	2.5
81	72	2	20.6	1	2	150	1700	50	205	1	86	6	4	50	7	28	57	15.4	2	16.8	5
82	66	2	25.4	1	1	50	700	40	130	1	87	2	2	10	5	28	58	8.8	2	16.2	2.5
83	65	1	23.9	1	1	100	1100	40	110	1	100	6	8	77	5	29	59	9	2	16.2	2.5
84	68	1	26	1	1	50	2200	40	165	1	77	8	9	89	7	29	59	15.4	2	16.8	5.2
85	75	2	25.7	1	2	100	1800	40	160	1	83	2	5	48	5	29	59	9	2	16.2	2.6
86	69	1	26.4	1	1	300	2400	35	120	1	74	6	5	60	5	29	60	10.2	2	16.2	2.6
87	73	2	23.5	1	1	150	1600	35	120	1	99	8	2	72	5	29	60	10.7	2	16.2	2.6
88	72	2	17.9	1	1	150	2200	40	130	1	99	0	4	25	6	29	60	11	2	16.2	2.6
89	76	2	25.6	1	1	100	2200	45	152	1	81	2	6	35	6	29	60	11.2	2	16.2	2.6
90	71	1	17.4	1	2	100	1140	50	200	1	77	8	8	89	7	29	62	15.4	2	16.8	5.2
91	70	1	21.6	1	1	50	1700	35	120	1	85	8	6	74	6	29	64	11.2	2	16.2	2.6
92	71	2	27	1	1	100	1700	35	120	1	82	5	8	70	6	29	65	11.6	2	16.2	2.6
93	71	1	23.8	1	1	50	1600	40	165	1	84	6	5	39	7	30	66	15.4	2	16.8	5.2
94	74	2	25.4	1	1	50	1700	45	135	1	98	6	8	70	6	30	66	11.6	2	16.3	2.6
95	73	1	29.7	1	1	150	1000	15	115	1	90	3	6	56	6	30	67	11.6	2	16.3	2.6
96	76	1	33.2	1	1	150	1600	45	175	2	79	9	5	90	8	30	67	16	3	17.2	5.6
97	65	1	30.1	1	1	150	2100	45.4	155	2	80	5	5	59	7	30	67	15.5	2	16.8	5.2
98	82	1	22.9	1	1	200	1850	50	250	2	60	4	6	55	9	31	70	16	3	17.2	5.6
99	66	2	24.9	1	1	30	1700	50	160	2	79	4	6	51	9	31	71	16.2	3	17.3	5.8
100	73	1	27.4	1	2	200	2200	50	175	2	61	8	3	76	9	31	71	16.2	3	17.3	5.8
101	78	1	26	1	2	200	2200	55	179	2	90	4	6	52	10	31	71	16.2	3	17.3	5.8
102	70	1	26.9	1	1	100	1100	40	170	2	78	7	3	76	10	31	74	16.2	3	17.5	6
103	81	1	27.1	1	1	300	1700	50	170	2	77	4	1	30	10	31	60	16.3	3	17.5	6.2
104	73	2	22.3	1	1	50	1200	50	280	2	83	6	4	49	10	40	60	16.3	3	17.7	6.2
105	72	2	26	1	2	150	2100	50	160	2	64	6	6	50	7	40	60	15.5	3	16.8	5.2
106	79	1	23.4	2	1	50	2100	45	179	2	67	6	4	70	10	40	61	16.4	3	17.8	6.2
107	72	1	26.7	1	1	100	1900	50	155	2	87	8	1	76	7	41	61	15.5	3	16.8	5.2
108	67	2	25.8	1	1	100	1600	45	150	2	68	3	3	20	10	41	62	16.5	3	18.1	6.3
109	76	1	23	1	1	100	1100	50	223	2	76	2	9	81	10	41	64	16.5	3	16.9	5.3
110	78	2	21.5	2	1	100	2200	50	260	2	85	5	8	76	11	41	65	16.5	3	16.9	5.3
111	70	2	24.9	1	1	50	1100	45	185	2	67	8	4	70	11	41	66	16.5	3	16.9	5.4
112	73	1	23.1	1	1	100	1600	45	180	2	67	2	5	43	11	42	66	16.5	3	17	5.4
113	66	2	25.5	1	1	50	1200	45	185	2	78	6	3	52	11	42	67	16.5	3	17.1	5.5
114	72	1	21.5	1	1	100	1700	45	173	2	75	3	4	38	11	42	80	16.5	3	17.2	5.5
115	66	1	18.4	1	1	100	1600	50	210	2	80	5	8	77	12	37	81	16.5	3	17.2	5.5
116	73	1	21.5	1	1	200	1200	50	165	2	70	6	4	76	12	37	81	16.5	3	17.2	5.5
117	77	2	29.3	1	1	100	1700	45	147	2	77	8	6	71	12	37	81	16.5	3	17.2	5.5
118	75	1	36.9	1	1	150	1700	40	170	2	100	8	4	68	12	37	82	16.5	3	17.2	5.6
119	68	1	22.5	1	1	100	1700	42.5	172	2	61	5	2	79	12	37	82	16.6	3	17.2	5.6
120	77	1	23.2	1	2	150	2300	55	175	2	73	8	8	79	12	37	83	16.6	3	17.3	5.8
121	65	1	22.9	1	1	300	1650	50	223	2	66	8	7	85	13	37	83	16.7	3	17.3	5.8
122	66	1	22.9	1	1	100	1800	50	225	2	86	4	1	32	13	38	83	16.7	3	17.3	5.8
123	67	1	23.8	1	1	200	1100	50	218	2	76	5	8	60	14	38	119	16.8	3	17.5	6
124	77	2	24.2	1	1	100	1600	45	155	2	86	5	8	78	7	38	123	15.5	3	16.9	5.2
125	70	1	22.8	1	2	100	1200	50	160	2	72	8	8	86	7	39	124	15.5	3	16.9	5.2
126	71	2	26.1	1	1	100	1600	50	165	2	64	4	6	51	15	39	124	16.9	3	17.5	6.2
127	76	2	20	1	1	200	2500	45	148	2	81	6	6	19	16	39	124	16.9	3	17.7	6.2
128	69	1	20.6	1	1	100	1200	45	185	2	75	2	8	77	16	39	134	17	4	20.3	9
129	67	1	25.3	1	1	150	1600	50	242	2	68	6	4	50	17	39	135	17	4	20.3	9.1
130	76	1	22.9	1	2	400	1700	55	175	2	86	4	4	30	17	39	140	17	4	20.3	9.1
131	71	2	23.6	1	2	200	2400	40	160	2	78	8	7	70	7	39	145	15.6	3	16.9	5.3
132	67	2	21.3	1	1	200	1350	50	283	2	65	6	7	65	18	39	155	17.2	4	20.6	7.2
133	65	1	20.7	1	1	200	1600	45	175	2	76	8	8	80	19	39	161	17.2	5	20.6	7.2
134	81	1	22.6	1	1	50	1100	50	235	2	73	5	4	56	20	39	171	17.4	5	21.1	7.3
135	70	1	21.3	1	1	100	1100	50	221	2	76	3	5	60	23	40	94	17.4	5	21.3	7.4
136	68	2	25.7	1	1	100	1100	45	150	2	81	8	4	71	27	40	94	17.4	5	22.3	7.5
137	75	2	24.9	1	2	100	1100	60	185	2	74	6	6	55	13	40	94	17.5	5	22.6	7.5
138	74	1	25.8	1	1	100	1600	45	175	2	78	1	6	30	13	40	96	17.6	4	19.4	6.3
139	75	1	26.7	1	2	150	1900	50	155	2	99	5	4	55	8	40	96	15.8	3	16.9	5.3
140	68	1	20.5	1	1	100	1700	45	185	2	85	8	5	76	14	40	97	17.6	4	19.4	6.5
141	72	1	25.1	1	1	50	1700	45	179	2	82	4	5	55	15	40	97	17.6	4	20	8.2
142	77	2	26.6	1	1	100	1700	45	145	2	74	2	5	44	16	40	102	17.7	4	20	8.3
143	66	2	25.7	1	2	300	2700	55	180	2	76	6	1	51	16	41	102	17.7	4	20.1	8.3
144	67	1	36.9	1	1	200	1700	50	220	2	79	10	7	76	17	41	119	17.7	4	20.2	8.3
145	70	1	26.6	1	2	400	1700	50	155	2	61	6	2	55	8	45	123	15.8	3	16.9	5.4
146	65	1	23.3	2	2	300	2700	50	160	2	62	5	2	46	8	45	124	15.9	3	17	5.4
147	71	1	25.8	1	2	200	1800	50	172	2	85	6	6	52	17	45	124	17.8	4	20.3	9
148	75	2	28.9	1	2	200	1020	60	180	2	76	6	5	43	18	46	124	17.8	4	20.3	9.1
149	66	1	26	1	1	50	1600	50	235	2	63	2	6	55	19	46	134	21.3	4	20.3	9.1
150	75	1	23.1	1	1	100	2300	45	175	2	62	9	7	92	20	46	135	22	4	20.6	7.2
151	65	2	25.4	1	1	100	1100	45	150	2	71	4	8	72	23	46	140	22.2	5	20.6	7.2
152	65	2	22.1	1	1	100	2300	45	150	2	71	1	5	40	7	47	145	22.3	5	21.1	7.3
153	83	2	26.1	1	1	200	1100	45	190	2	88	5	5	36	7	47	155	22.4	3	18.2	4.3
154	76	1	22.9	1	2	100	1250	50	160	2	87	5	2	43	8	48	161	16	3	17.1	5.5
155	70	2	30.4	1	1	100	1300	50	160	2	74	7	4	62	7	48	171	22.4	3	18.2	4.3
156	71	2	22.1	1	2	300	1300	40	160	2	64	3	6	55	8	48	179	16	3	17.2	5.5
157	66	1	23.9	2	1	150	2900	50	170	2	99	2	3	29	7	48	94	22.6	3	18.2	4.3
158	73	2	28.9	1	1	100	1700	50	163	2	67	5	4	42	7	49	94	23	3	18.2	4.3
159	68	2	30.4	1	1	100	1600	50	265	2	89	8	4	72	7	49	94	23.2	3	18.2	4.4
160	71	2	33.1	1	1	200	2100	45	148	2	85	5	10	95	7	49	94	25.1	3	18.2	4.4
161	68	2	30.9	1	1	100	1200	45	190	2	85	1	8	72	7	50	94	26.2	3	18.2	4.5
162	76	2	22.9	1	1	150	2700	45	185	2	61	6	5	56	7	50	96	26.2	3	18.4	4.5
163	68	2	19.5	1	2	400	3100	55	179	2	69	6	6	55	7	50	96	26.3	3	18.4	4.5
164	71	1	20.7	1	1	80	1300	50	221	2	86	9	7	98	7	50	87	22.6	3	18.5	4.6
165	77	2	23.6	1	1	20	1700	45	145	2	77	4	8	71	7	50	87	23	3	18.5	4.6
166	69	1	22.2	1	1	150	1600	45	180	2	83	8	8	79	7	50	88	23.2	3	18.5	4.8
167	72	2	29.4	1	2	100	1100	55	180	2	85	5	5	40	7	51	88	25.1	3	18.6	5
168	71	1	23.8	1	2	100	1600	50	170	2	89	5	3	55	7	51	89	26.2	3	18.6	5
169	73	1	19	1	2	200	1000	50	173	2	61	7	6	78	6	51	90	26.2	3	18.7	5
170	79	2	26.6	1	1	100	1100	50	268	2	79	8	5	75	6	52	90	26.3	3	18.9	5.2
171	65	1	21.5	1	1	100	1600	50	210	2	90	7	5	55	6	52	90	26.6	3	19	5.2
172	66	2	19.8	1	2	50	1700	45	150	2	100	9	5	87	6	52	90	27	3	19	5.2
173	75	2	25.5	1	1	300	2000	50	160	2	83	4	0	30	6	54	91	27.1	3	19.2	6.3
174	74	1	20.1	2	2	200	2300	50	175	2	86	7	5	62	6	54	92	27.9	3	19.2	6.3
175	70	2	20.6	1	1	200	1100	45	150	2	82	4	5	46	6	55	94	22	4	19.2	6.3
176	77	1	26.4	1	2	200	1600	50	170	2	77	8	4	71	6	56	94	22.2	4	19.3	6.3
177	65	1	27.9	2	1	200	2100	45	180	2	84	5	7	64	13	56	94	22.3	4	19.3	6.3
178	75	1	25.4	1	1	150	1100	50	225	2	77	8	8	79	7	57	94	22.4	4	19.4	6.3
179	71	1	21.5	1	2	200	1500	50	155	2	88	8	5	89	8	57	94	16	3	17.2	5.5
